# Supplementary material for: Rational design of hammerhead ribozymes as CD93 silencing tools for vascular diseases
Source: Mol Ther Nucleic Acids. 2026 May 25;37(2):102968. doi: 10.1016/j.omtn.2026.102968 (PMC13264038; doi:10.1016/j.omtn.2026.102968)
Supplement: Document S1. Figures S1–S3 and Table S1 [file mmc1.pdf]

OMTN, Volume *37*

Supplemental information

Rational design of hammerhead ribozymes

as CD93 silencing tools for vascular diseases

Cosimo Damiano Perrone, Luisa Raucci, Sara Papini, Gian Marco Tosi, Federico Galvagni, Massimo Olivucci, Danny Incarnato, and Maurizio Orlandini

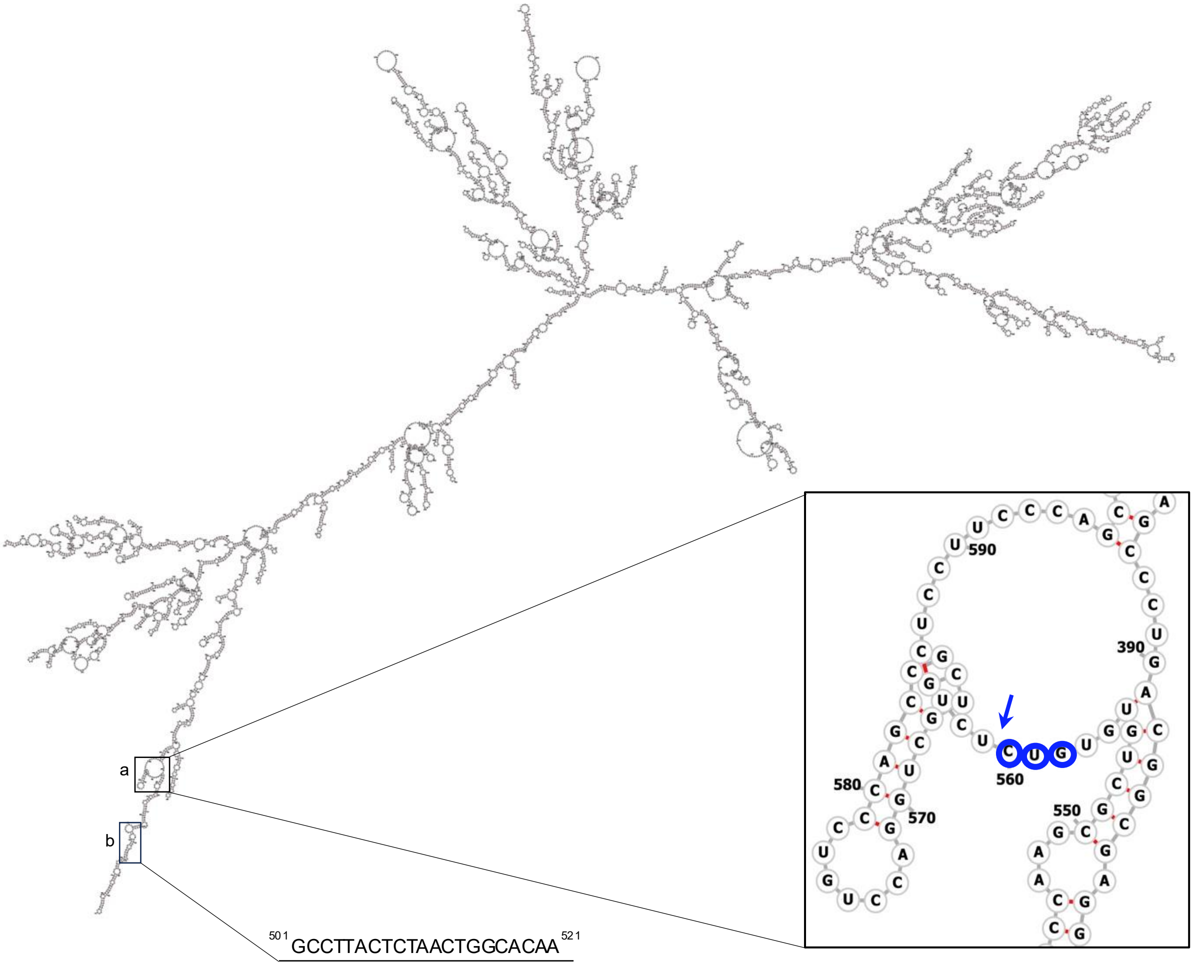

Fig. S1. **Selection of the hhRz target motif within the CD93 mRNA transcript**. The predicted secondary structure (RNAfold) of the full-length CD93 mRNA (6681 nucleotides) is shown. Inset (a) shows the loop containing the selected cleavage site (blue arrow) and the NUH triplet highlighted in blue. Inset (b) displays the CD93 region that pairs with the shRNA (TRCN0000029085) previously used to silence CD93 expression in human ECs.

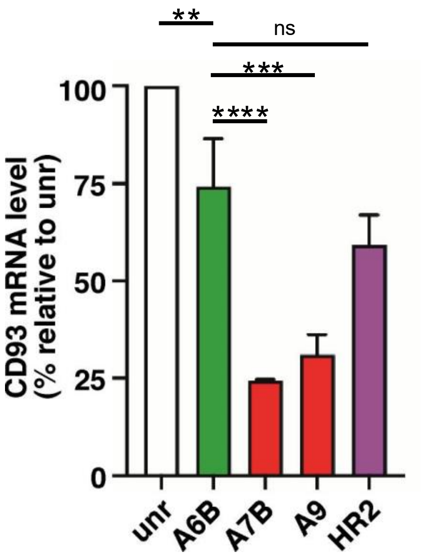

Fig. S2. **RT-qPCR analysis of CD93 mRNA levels following hhRz expression**. Primary HUVECs were transduced with lentiviral particles expressing either an unrelated RNA (unr) or the indicated ch-hhRz construct. Cells were selected with puromycin for 72 h. CD93 mRNA levels were quantified by RT-qPCR, normalized to β-actin, and expressed as percentages relative to control cells (unr). $^{**}P < 0.01$ $^{***}P < 0.001$ $^{****}P < 0.0001$, ns (not significant); one-way ANOVA.

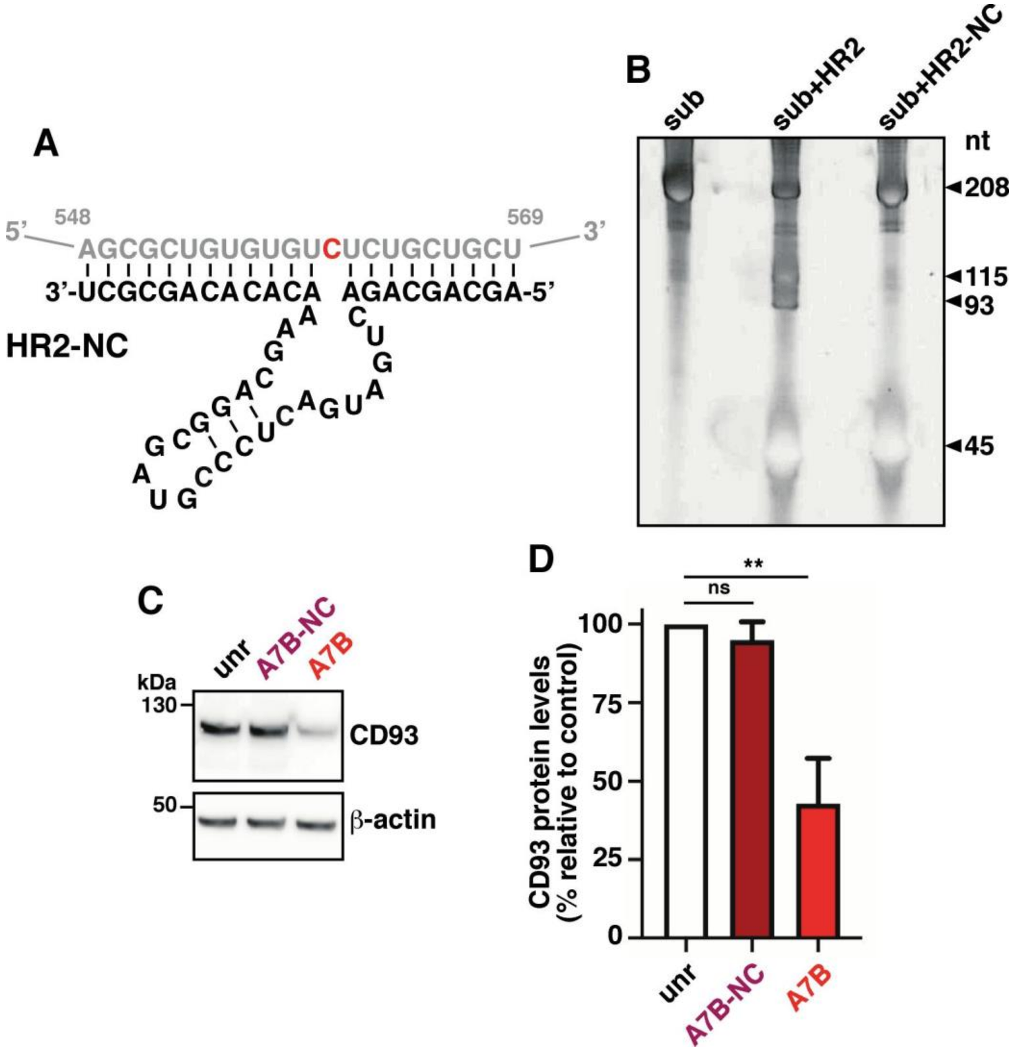

Fig. S3. **Ribozyme-mediated CD93 silencing requires catalytic activity**. A: Secondary structure of the synthetic ribozyme-substrate complex targeting the 558-GUC-560 sequence located within the coding region of CD93 mRNA. HR2-NC carries two mutations in the catalytic core. The cleavage site is indicated in red. Watson-Crick base pairs are shown as dotted lines. B: Denaturing gel electrophoresis showing the cleavage activity of HR2 and HR2-NC on a synthetic CD93 RNA substrate. The substrate (sub) is 208 nucleotides in length and corresponds to nucleotides 468-675 of the full-length CD93 mRNA. Cleavage at the target site (558-GUC-560) generates two fragments of 93 and 115 nucleotides. The control lane shows the untreated RNA substrate (sub). Fragment sizes are indicated in nucleotides (nt). C: HEK 293 cells were co-transfected with a CD93 expression construct and plasmids encoding ch-A7B or ch-A7B-NC, the latter carrying mutations in the catalytic core analogous to HR2-NC. 72 h post-transfection, cell lysates were analyzed by Western blot analysis using anti-CD93 and anti-β-actin antibodies. D: Quantification of CD93 protein levels from experiments performed as in C. Values were normalized to β-actin and expressed as percentages relative to unrelated transfected control cells (unr). $**P < 0.01$, ns (not significant); paired *t*-test.

Table S1. Primer sequences used for hhRz cloning into the pLKO.1 vector.

Oligo	Sequence
A6B fw	ccggtGGCTCTTCTCAGAGCCAAAAACAGGAGAGGCTGATGAGTCCGGTAGCGGACGAAACCCAGCCTAAAAAGGCTCTTCTCAGAGCCTTTTTg
A6B rev	aattcAAAAAGGCTCTGAGAAGAGCCTTTTTAGGCTGGGTTTCGTCCGCTACCGGACTCATCAGCCTCTCCTGTTTTTGGCTCTGAGAAGAGCCa
A4B fw	ccggtGGCTCTTCTCAGAGCCAAAAAGACCTATTTGCTGATGAGTCCGGTAGCGGACGAAACAACAAATCAAAAAGGCTCTTCTCAGAGCCTTTTTg
A4B rev	aattcAAAAAGGCTCTGAGAAGAGCCTTTTTGATTTGTTGTTTCGTCCGCTACCGGACTCATCAGCAAATAGGTCTTTTTGGCTCTGAGAAGAGCCa
A7B fw	ccggtGGCTCTTCTCAGAGCCAAAAAGCTTTAAAATCTGATGAGTCCGGTAGCGGACGAAACAACTTTCAAAAAAGGCTCTTCTCAGAGCCTTTTTg
A7B rev	aattcAAAAAGGCTCTGAGAAGAGCCTTTTTTGAAAGTTGTTTCGTCCGCTACCGGACTCATCAGATTTTAAAGCTTTTTGGCTCTGAGAAGAGCCa
A11 fw	ccggtGGCTCTTCTCAGAGCCAAAAAGTTCCAACTCTGATGAGTCCGGTAGCGGACGAAAAAACTGGAGAAAAAGGCTCTTCTCAGAGCCTTTTTg
A11 rev	aattcAAAAAGGCTCTGAGAAGAGCCTTTTTCTCCAGTTTTTTCGTCCGCTACCGGACTCATCAGAGTTGGAACTTTTTGGCTCTGAGAAGAGCCa
A9 fw	ccggtGGCTCTTCTCAGAGCCAAAAAAAGGCTATTACTGATGAGTCCGGTAGCGGACGAAAAATGTAAGAAAAAAGGCTCTTCTCAGAGCCTTTTTg
A9 rev	aattcAAAAAGGCTCTGAGAAGAGCCTTTTTTCTTACATTTTTCGTCCGCTACCGGACTCATCAGTAATAGCCTTTTTTTGGCTCTGAGAAGAGCCa
A10 fw	ccggtGGCTCTTCTCAGAGCCAAAAAAAAACAATCTCTGATGAGTCCGGTAGCGGACGAAAACCATAGCAAAAAAGGCTCTTCTCAGAGCCTTTTTg
A10 rev	aattcAAAAAGGCTCTGAGAAGAGCCTTTTTTGCTATGGTTTTCGTCCGCTACCGGACTCATCAGAGATTGTTTTTTTTTGGCTCTGAGAAGAGCCa
A12 fw	ccggtGGCTCTTCTCAGAGCCAAAAAGGCCACCCTGATGAGTCCGGTAGCGGACGAAAAGGCAGAGAAAAAGGCTCTTCTCAGAGCCTTTTTg
A12 rev	aattcAAAAAGGCTCTGAGAAGAGCCTTTTTCTCTGCCTTTTCGTCCGCTACCGGACTCATCAGGGTGGCCTTTTTGGCTCTGAGAAGAGCCa
HR2 fw	ccggtGGCTCTTCTCAGAGCCAAAAAGCAGCAGACTGATGAGTCCGGTAGCGGACGAAACACACAGCGCTAAAAAGGCTCTTCTCAGAGCCTTTTTg
HR2 rev	aattcAAAAAGGCTCTGAGAAGAGCCTTTTTAGCGCTGTGTGTTTCGTCCGCTACCGGACTCATCAGTCTGCTGCTTTTTGGCTCTGAGAAGAGCCa
A7B-w fw	ccggtGCTTTAAAATCTGATGAGTCCGGTAGCGGACGAAACAACTTTCATTTTTg
A7B-w rev	aattcAAAAATGAAAGTTGTTTCGTCCGCTACCGGACTCATCAGATTTTAAAGCa
A7B-NC fw	ccggtGGCTCTTCTCAGAGCCAAAAAGCTTTAAAATCTGATGACTCCCGTAGCGGACGAAACAACTTTCAAAAAAGGCTCTTCTCAGAGCCTTTTTg
A7B-NC rev	aattcAAAAAGGCTCTGAGAAGAGCCTTTTTTGAAAGTTGTTTCGTCCGCTACGGGAGTCATCAGATTTTAAAGCTTTTTGGCTCTGAGAAGAGCCa
