# Supplementary material for: Rational design of hammerhead ribozymes as CD93 silencing tools for vascular diseases
Source: Mol Ther Nucleic Acids. 2026 May 25;37(2):102968. doi: 10.1016/j.omtn.2026.102968 (PMC13264038; doi:10.1016/j.omtn.2026.102968)
Supplement: Document S2. Article plus supplemental information [file mmc2.pdf]

Molecular Therapy
Nucleic Acids
Original Article

Rational design of hammerhead ribozymes as CD93 silencing tools for vascular diseases

Cosimo Damiano Perrone,[1] Luisa Raucci,[1] Sara Papini,[1] Gian Marco Tosi,[2] Federico Galvagni,[1] Massimo Olivucci,[1] Danny Incarnato,[3] and Maurizio Orlandini[1]

[1]Department of Biotechnology, Chemistry and Pharmacy, University of Siena, 53100 Siena, Italy; [2]Department of Medicine, Surgery and Neuroscience, Ophthalmology Unit, University of Siena, 53100 Siena, Italy; [3]Department of Molecular Genetics, Groningen Biomolecular Sciences and Biotechnology Institute (GBB), University of Groningen, 9747 AG Groningen, the Netherlands

While targeting angiogenesis represents a key therapeutic strategy in several pathological contexts, it comes with significant challenges associated with therapeutic efficacy and drug resistance. Therefore, developing novel, more effective and durable therapeutic modalities is paramount. We have previously reported that antibody-mediated inhibition of CD93, a vascular endothelial cell surface glycoprotein, can inhibit angiogenesis. Here, we describe a novel strategy to efficiently inhibit CD93 expression in cells, based on the targeted degradation of CD93 mRNA using *trans*-acting hammerhead ribozymes. To pinpoint single-stranded regions in CD93 mRNA that are amenable to ribozyme targeting in living cells, we performed RNA secondary structure mapping via targeted DMS-MaPseq analysis. Next, since exogenous hammerhead ribozymes are easily degraded and lose catalytic activity when expressed in living cells, we developed a novel scaffold RNA based on short stems from the 3′ UTR of histone mRNAs to stabilize the active ribozyme structure and promote the CD93 cleavage under physiological conditions. Ectopic expression of these engineered ribozymes in primary endothelial cells resulted in efficient inhibition of CD93 expression, cell migration, and formation of tube-like structures in functional assays. Collectively, our data provides the proof-of-concept for the use of ribozyme-based therapeutics for the treatment of neovascular pathologies.

INTRODUCTION

Angiogenesis, the process by which new blood vessels form from pre-existing ones, is essential for normal development and adult physiology, and its deregulation is associated with a wide range of pathological conditions.[1] Over the past decades, several antiangiogenic drugs have been developed to prevent the progression of different neovascular diseases, including cancer and ocular disorders.[2] However, because multiple converging pathways can promote angiogenesis, its inhibition often results in weak clinical outcomes and the development of drug resistance. Therefore, the discovery of new antiangiogenic targets is paramount.[2]

We and others have previously shown that silencing of CD93, a glycosylated transmembrane protein prevalently expressed on endothelial cells (ECs), leads to impaired EC adhesion, migration, and formation of vessel sprouts, which are critical steps during angiogenesis.[3,4] The CD93 receptor exerts its proangiogenic activity by directly binding extracellular matrix proteins, such as Multimerin-2 and IGFBP7, and modulating the function of different transmembrane proteins, including β-dystroglycan, VEGFR-2, β1-integrin, and VE-cadherin.[5–8] Effectively, disruption of the CD93/Multimerin-2 interaction reduces EC adhesion and migration and blockade of the CD93/IGFBP7 pathway normalizes tumor vasculature promoting drug delivery and immunotherapy).[9,10] Hence, CD93 represents an ideal target for antiangiogenic treatment also because in contrast to pathological blood vessels, it is weakly expressed on ECs of normal resting blood vessels.[11,12] Accordingly, targeting CD93 has been proposed as a viable antiangiogenic therapeutic strategy for the treatment of cancer and other diseases.[13–15]

In this work, we sought to develop a new therapeutic post-transcriptional gene silencing strategy to inhibit the function of the CD93 receptor in ECs, by designing hammerhead ribozymes (hhRzs). Targeting CD93 at the mRNA level may offer potential advantages compared with antibody-based strategies. While therapeutic antibodies can effectively block ligand-receptor interactions, RNA-based approaches reduce the production of the receptor itself, thereby preventing the activation of multiple downstream signaling pathways.[16] In addition, catalytic RNAs such as hhRzs provide sequence-specific cleavage of target transcripts through an intrinsic catalytic activity, enabling precise post-transcriptional gene silencing. Indeed, hhRzs are short RNA sequences that catalyze the cleavage of target polyribonucleotides in a protein-independent fashion.[17] First discovered as self-cleaving sequences in plant infectious viroids,[18] hhRzs were next developed as *trans*-acting sequences to cleave any desired RNA molecule by taking advantage of their conserved secondary structure.[19] *Trans*-cleaving hhRzs are engineered to contain a conserved catalytic core able to cleave RNA substrates at the level

Received 19 December 2025; accepted 21 May 2026;
https://doi.org/10.1016/j.omtn.2026.102968.

Correspondence: Maurizio Orlandini, Ph.D., Department of Biotechnology, Chemistry and Pharmacy, University of Siena, Via A. Moro, 2, 53100 Siena, Italy.
E-mail: maurizio.orlandini@unisi.it

Published by Elsevier Inc. on behalf of The American Society of Gene and Cell Therapy.
This is an open access article under the CC BY license (http://creativecommons.org/licenses/by/4.0/).

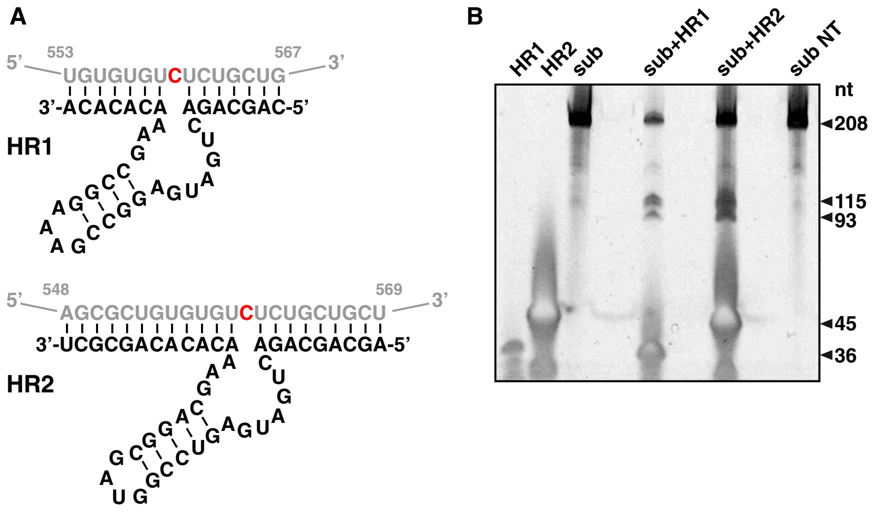

Figure 1. hhRbzs specifically targeting CD93 mRNA
(A) Secondary structures of synthetic ribozyme-substrate complexes targeting the 558-GUC-560 sequence located within the coding region of CD93 mRNA. HR1 and HR2 differ in their catalytic core sequences. Numbers above the structures indicate the positions of base pairing between the ribozymes (black) and the human CD93 mRNA sequence (gray). The cleavage site is indicated in red. Watson-Crick base pairs are shown as dotted lines. (B) *Trans*-cleaving activity of HR1 and HR2 on a synthetic CD93 RNA substrate. Denaturing gel electrophoresis shows HR1 and HR2 cleavage activity after 2 h of incubation at 37°C. The synthetic RNA substrate (sub) is 208 nucleotides long, corresponding to nucleotides 468–675 of the full-length CD93 mRNA. Cleavage at the target sequence (558-GUC-560) generates two fragments of 93 and 115 nucleotides. Control lanes show ribozymes (HR1 and HR2) and the target RNA (sub) as freshly prepared samples, as well as the substrate RNA incubated for 2 h in the absence of ribozymes (sub NT). RNA fragment lengths are indicated in nucleotides (nt).

of NUH triplets (where N is any base and H is A, C, or U) and two lateral arms complementary to an accessible region of a target RNA molecule.[20] HhRzs represent attractive tools for the development of RNA-based therapeutic agents due to their small size, easy design, specificity of target recognition, and precise cleavage, with no detectable off-target effects.[21] Nevertheless, several challenges yet remain to be addressed to efficiently exploit engineered hhRzs for gene silencing in living cells, such as delivery to target cells, poor enzymatic activity, and high exogenous RNA instability in the cellular environment.[22]

Herein, we designed hhRzs targeting CD93 mRNA and validated the most efficient candidate in human primary ECs. To further increase the stability of hhRzs in living cells, we employed a novel RNA scaffold, based on the stem-loop sequence found at the 3′ end of histone mRNAs,[23] thus generating new RNA-based tools to knock down the expression of CD93 in physiological conditions.

RESULTS

Design and *in vitro* validation of hhRzs targeting CD93 mRNA

To test whether *trans*-acting hhRzs could constitute a viable strategy to induce the degradation of the CD93 mRNA, we first selected a candidate hhRz cleavage site in the proximity of an shRNA target sequence that we had previously shown to efficiently silence CD93 expression by RNA interference.[3] The target site at position 560 of the full-length CD93 mRNA was predicted to be localized within a largely accessible region, based on minimum free energy (MFE) secondary structure prediction (Figure S1). Since several strategies have been proposed to enhance the *trans*-cleaving activity of hhRzs, we designed two ribozymes with distinct catalytic core: HR1, containing the evolutionarily conserved catalytic core[24] and HR2, harboring a variant core known to retain strong activity under physiological conditions.[22] In each ribozyme, 5′ and 3′ CD93-antisense flanks were introduced to ensure molecular recognition of the accessible region within the human CD93 mRNA (Figure 1A; Table 1). To verify the ability of HR1 and HR2 to cleave the CD93 mRNA, we first performed an *in vitro* cleavage assay using a synthetic RNA substrate corresponding to the CD93 mRNA region spanning nucleotides 468–675 (208 nucleotides in length). As expected, hhRz-mediated cleavage generated two RNA fragments of 93 and 115 nucleotides (Figure 1B). As both ribozymes displayed comparable efficiencies at 37°C, we decided to carry out subsequent in cell experiments using the HR2 catalytic core, which has previously been shown to maintain robust cleavage activity at low $MgCl_2$ concentrations, comparable to the intracellular environment.[22]

Strategies to enhance the intracellular stability of hhRz RNA

Since *trans*-acting hhRzs are prone to degradation when expressed in living cells,[25] we devised an RNA scaffold designed to protect the ribozyme from rapid digestion by cellular RNases. To this end, we flanked both ends of HR2 with the 3′ stem-loop sequence of canonical histone mRNAs.[23] This 16-nucleotide stem-loop structure is composed of a four-nucleotide loop and a six-base-pair stem, separated from HR2 by a short stretch of 4–5 adenines (Figure 2A). To assess the potential activity of this chimeric HR2 (ch-HR2) molecule, we first analyzed its secondary structure. The use of a synthetic RNA substrate corresponding to the CD93 mRNA region spanning nucleotides 468–732 (265 nucleotides long), showed comparable cleavage efficiencies between ch-HR2 and HR2 lacking the added stem-loops (Figure 2B). To further assess whether ch-HR2 could downregulate CD93 expression in a physiological cellular context, we cloned ch-HR2 into a lentiviral vector to enable efficient delivery of the exogenous construct and sustained expression of the catalytic hhRz in living cells.[26] Human umbilical vein ECs (HUVECs) were transduced as described earlier, and cell extracts were subsequently analyzed by immunoblotting. This analysis revealed an approximately 33%

Table 1. Nucleotide sequences of hhRzs specific for CD93 mRNA

hhRz	Stem I	Stem II (catalytic core)	Stem III
HR1	567-CAGCAGA	CUGAUGAGGCCGAAA–GGCCGAA	ACACACA-553
HR2	569-AGCAGCAGA	CUGAUGAGUCCGGUAgcGGACGAA	ACACACAGCGCU-548
HR2-NC	569-AGCAGCAGA	CUGAUGAcUCCcGUAGCGGACGAA	ACACACAGCGCU-548

Stem I and stem III contain the annealing sequences targeting CD93 adjacent to a NUH triplet, where N represents any nucleotide, U is uridine, and H represents any nucleotide except guanosine. Differences between HR1 and HR2 within the catalytic core are indicated (base substitutions, underlined; insertions, lowercase). HR2-NC was used as a negative control for catalytic activity and carries two point mutations in the catalytic core (lowercase).

reduction in CD93 protein levels compared with lysates from ECs transduced with a lentivirus expressing an unrelated RNA (Figures 2C and 2D). These results suggest that the inclusion of histone stem-loop elements does not impair the ability of *trans*-acting hhRzs to interact with and cleave their target RNA sequence.

Identification and validation of hhRz accessible regions in human CD93 mRNA

While promising, these results revealed only a suboptimal reduction of CD93 levels. As efficient targeting by *trans*-acting hhRzs requires base pairing with the target RNA, we investigated whether mapping the CD93 mRNA secondary structure in cells could help identify more accessible target sites. To obtain a map of structural accessibility along the CD93 mRNA, we performed dimethyl sulfate (DMS) probing coupled with targeted mutational profiling analysis and sequencing (DMS-MaPseq) (Figure 3A), as previously described.[27] The identified regions were then classified according to their accessibility score (high, mid, or low; see materials and methods) and within these regions, we searched for potential NUH triplets required for hhRz-mediated cleavage. For the selected catalytic core, the most favorable cleavage triplet is GUC,[22] which was rarely found within regions displaying the highest accessibility scores. Nonetheless, we identified two potential target sites located in highly accessible regions (positions: 3,139 and 4,903) (Table 2). For comparison, we also selected one site with a low accessibility score (position: 4,443). In addition, we investigated four targets containing the alternative UUC triplet, as this motif was the most frequently observed, and selected two sites within highly accessible regions (positions: 2,735 and 3,815) as well as two additional sites located in regions with mid (position: 3,936) and low (position: 5,265) accessibility scores, respectively.

To rapidly assess the efficiency of *trans*-acting hhRzs targeting these seven sites, we first cloned the full-length CD93 mRNA into a eukaryotic expression vector and co-transfected it with ch-hhRz constructs, containing the 5′ and 3′ histone mRNA stem-loop elements, into human embryonic kidney (HEK) 293 cells, which lack endogenous CD93 expression.[3] Western blot analyses of the transfected cells revealed a clear correlation between higher accessibility scores and increased ribozyme-mediated knockdown efficiency (Figures 3B and 3C), confirming that regions predicted to be more accessible are indeed more susceptible to hhRz cleavage. Notably, the A4B and A7B ch-hhRz constructs, whose targets contained the optimal GUC triplet, displayed higher silencing efficiency than constructs A9 and A10, whose targets contained the suboptimal UUC triplet. These results indicate that both target structural accessibility and cleavage triplet identity play key roles in determining ribozyme efficiency. Moreover, the catalytically inactive A7B-NC ch-hhRz mutant, carrying point mutations in the catalytic core (Figures S3A and S3B), failed to downregulate CD93 protein levels (Figures S3C and S3D). As these mutations preserve target binding via the antisense arms, this result indicates that CD93 silencing depends on catalytic activity rather than antisense-mediated inhibition.

To further confirm that ch-hhRz constructs could also cleave the endogenous CD93 mRNA under physiological conditions, we generated lentiviruses encoding the best-performing ribozymes for both triplets (A7B and A9), as well as for the less efficient ch-hhRz (A6B) as a control and used them to infect primary HUVECs. Consistent with our previous findings, ribozymes targeting regions predicted to be the most accessible led to a significant reduction in CD93 protein levels, as shown by western blot analyses (Figures 3D and 3E). mRNA levels were also reduced, as assessed by quantitative RT-PCR analysis (Figure S2). Furthermore, to evaluate the contribution of the histone stem-loop scaffold to ribozyme efficiency, we removed the stem-loop elements from the best-performing ch-hhRz (A7B), generating the construct A7B-w. Lentiviruses encoding A7B-w were used to infect HUVECs, and western blot analysis of CD93 levels revealed that removal of the stem-loop elements drastically reduced the silencing efficiency of the ribozyme, confirming that the introduced scaffold plays an important role in enhancing hhRz stability and activity in cells (Figure 3F). Together, these results demonstrate that our experimental strategy enables the rational identification of optimal hhRz target sites for efficient gene silencing in living cells.

CD93 knockdown by hhRz expression impairs EC function

Since we previously demonstrated that silencing CD93 expression by shRNAs markedly affects the migration and differentiation of human primary ECs,[3] we next investigated whether the ch-hhRz-mediated cleavage of CD93 could exert similar functional effects on EC behavior. To assess this, HUVECs were transduced with lentiviral vectors expressing the top-performing A7B ch-hhRz, and western blot analysis confirmed a marked reduction in CD93 protein levels compared with control (Figure 4A). Consistent with this decrease, the migratory ability of HUVECs was significantly impaired in

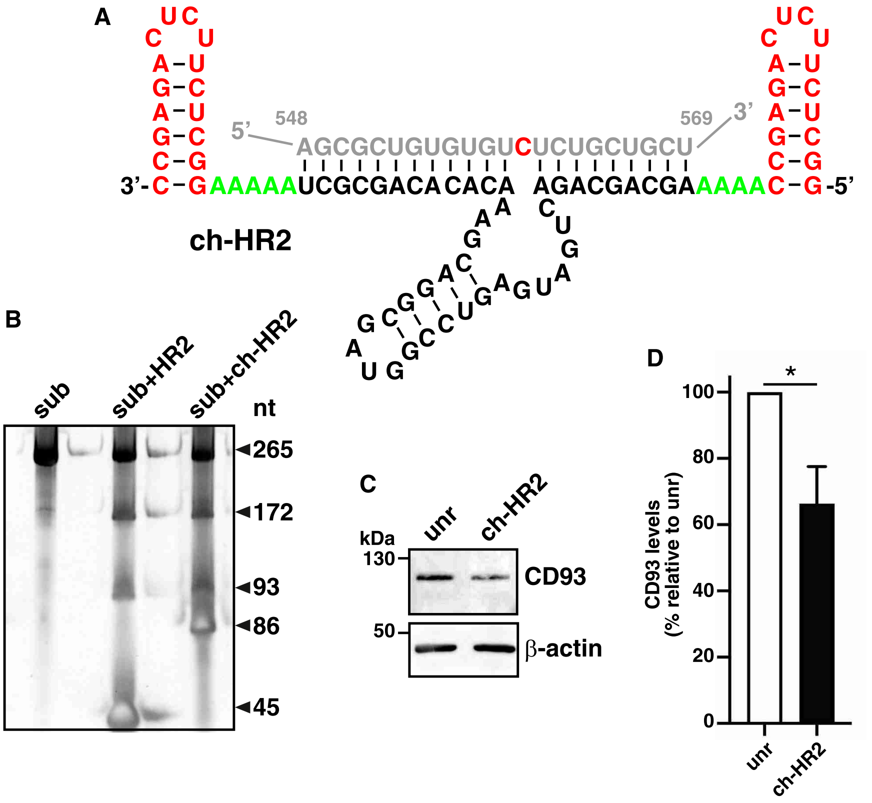

Figure 2. Structure and activity of the ch-HR2 chimera

(A) Sequence and predicted secondary structure of the HR2 ribozyme (black) fused with the histone 3′ end sequences (red) to form the chimeric construct (ch-HR2), which anneals to the human CD93 mRNA sequence (gray). A stretch of adenines is shown in green. Watson-Crick base pairs are indicated by dotted lines. (B) Denaturing gel electrophoresis showing the cleavage activity of HR2 and ch-HR2 after 3 h of incubation at 37°C. The synthetic RNA substrate (sub) is 265 nucleotides long and corresponds to nucleotides 468–732 of the full-length CD93 mRNA. Cleavage at the target site (558-GUC-560) generates two fragments of 93 and 172 nucleotides. Control lane shows the substrate RNA (sub) after 3 h of incubation at 37°C in the absence of ribozymes. The lengths of RNA fragments are indicated in nucleotides (nt). (C) HUVECs were transduced with lentiviral particles expressing ch-HR2 or an unrelated control (unr). Cell lysates were subjected to western blotting analysis using anti-CD93 and anti-β-actin antibodies, the latter serving as a loading control. (D) Quantification of CD93 protein levels from experiments performed as in (C). Values were normalized to β-actin and expressed as percentages relative to unrelated transduced control cells (unr). $^{*}p < 0.05$; paired *t* test.

wound healing assays because cells expressing A7B failed to efficiently close the wound gap within the same time frame as control RNA-expressing cells (Figures 4B and 4C). Similarly, in a tube formation assay on Matrigel, a substrate that supports EC attachment and differentiation, control-transduced HUVECs organized into interconnected tubular networks, whereas A7B-expressing cells formed poorly developed and fragmented structures, showing a significant reduction in both total tube length and branch number (Figures 4D and 4E). Together, these findings indicate that the newly designed scaffold RNA preserves the proper ribozyme conformation, enabling its *trans*-cleaving activity to effectively silence specific target genes under physiological conditions.

DISCUSSION

The discovery of novel endothelial markers and the identification of proangiogenic molecules are major goals in the development of effective antiangiogenic therapies. CD93 is a transmembrane protein that has recently emerged as a promising target for antiangiogenic treatment,[13,28] particularly in retinal vascular disorders such as age-related macular degeneration (AMD), in which the regression of choroidal neovessels is associated with vision recovery.[14] Although intravitreal anti-VEGF injections have revolutionized AMD treatment, a significant proportion of patients either fail to respond or require frequent intravitreal injections to prevent disease recurrence.[29] The permanent genetic ablation of chronically expressed proangiogenic factors in AMD patients could represent a valuable improvement over the current therapeutic approach, reducing the injection burden and potentially improving visual outcomes in non-responders. Importantly, the eye is an ideal organ for gene therapy because of its limited immune response and easy accessibility, which allows efficient delivery of genetic material to target cells.[30] Notably, nucleic-acid-based therapeutics have already been successfully used in clinical practice for ocular diseases, including the anti-VEGF aptamer pegaptanib and siRNA-based approaches targeting angiogenic pathways.[31,32] In this study, we developed a *trans*-acting hhRz designed to cleave the CD93 mRNA and demonstrated its ability to downregulate this pro-migratory receptor in proliferating ECs. This hhRz-based approach for CD93 silencing could serve as a valuable genetic tool for the treatment of AMD for several reasons. First, the hhRz can be efficiently delivered to choroidal ECs through adeno-associated virus (AAV) vectors, which are among the most widely used viral vectors in retinal gene therapy and have recently received FDA approval for clinical trials in AMD patients.[33] Importantly, several engineered AAV capsids with enhanced tropism for ECs have been developed. For example, AAV-BR1, identified through *in vivo* selection, preferentially transduces vascular ECs in the central nervous system and retina,[34] and improved derivatives such as AAV-BI30 further enhance endothelial targeting efficiency.[35] In addition, engineered capsids such as R100 have been optimized for ocular gene delivery and are currently being evaluated in clinical trials for retinal diseases, supporting the feasibility of AAV-mediated gene delivery in the eye.[36] Second, although

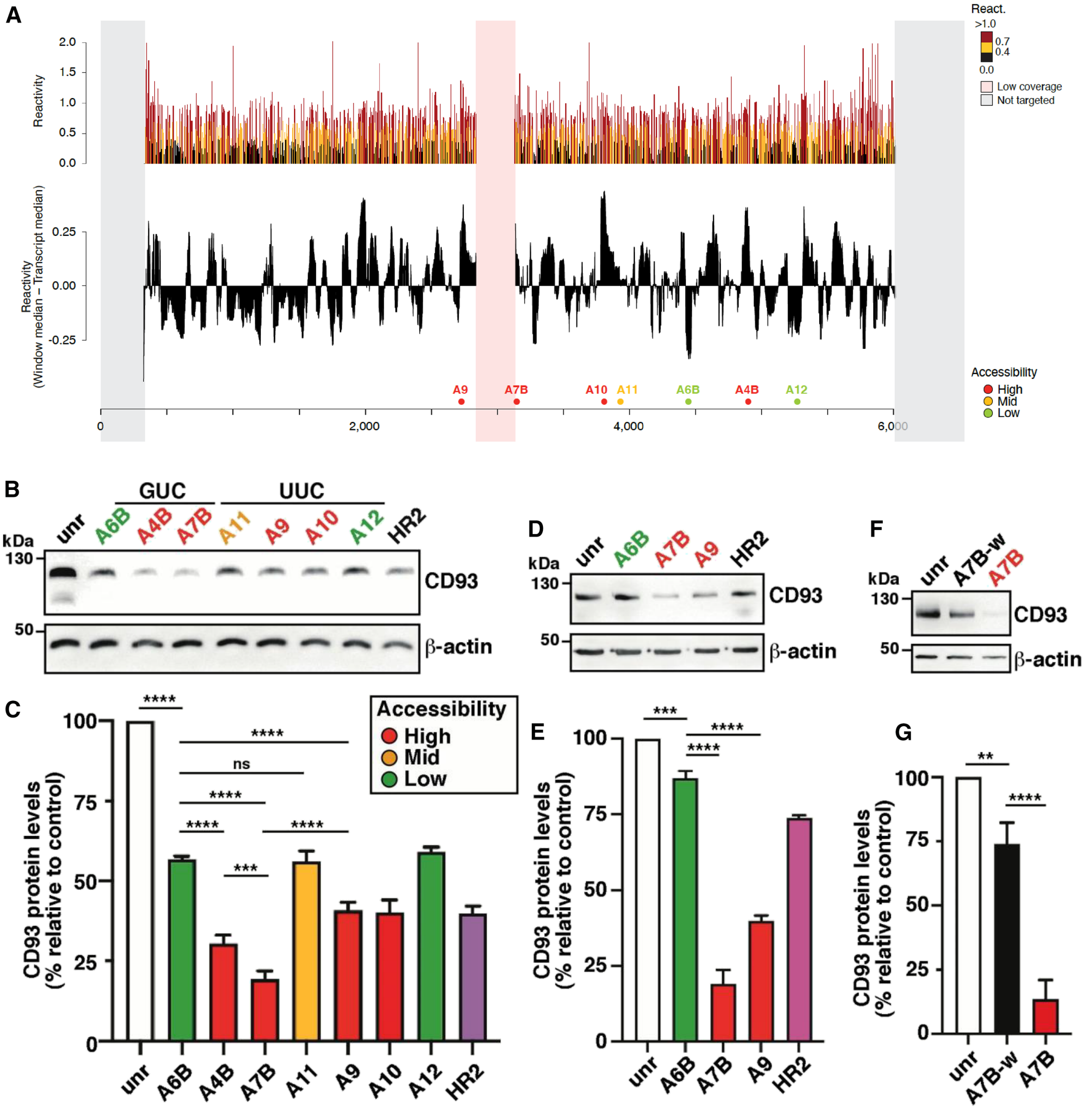

Figure 3. Selection of hhRz-accessible regions within the CD93 mRNA

(A) DMS-MaPseq analysis of CD93 mRNA in HUVECs. From top to the bottom, the figure shows per-base normalized DMS reactivities, the sliding-window median reactivity plotted relative to the median reactivity across the entire transcript, and the positions of the ribozyme target sites analyzed. Terminal regions not captured by the targeted analysis are shown in gray, while a region with low sequencing coverage is highlighted in pink. (B) HEK293 cells were co-transfected with the CD93 expression construct and plasmids encoding the indicated chimeric hhRzs. 72 h after transfection, cell lysates were subjected to western blot analysis using anti-CD93 and anti-β-actin antibodies. (C) Quantification of CD93 protein levels from experiments performed as in (B). Values were normalized to β-actin and expressed as percentages relative to unrelated-transfected control cells (unr). ***$p < 0.001$, ****$p < 0.0001$, ns (not significant); one-way ANOVA. (D) Western blot analysis of cell lysates from primary HUVECs transduced with lentiviral particles expressing either an unrelated (unr) or ch-hhRz RNAs. Cells were selected with puromycin for 72 h and analyzed using anti-CD93 and anti-β-actin antibodies. (E) Quantification of CD93 protein levels from experiments performed as in (D). Values were normalized to β-actin and expressed as percentages relative to control cells (unr). ***$p < 0.001$, ****$p < 0.0001$; one-way ANOVA. hhRzs are color-coded according to their accessibility scores, shown in the inset. (F) Western blot analysis of cell lysates from primary HUVECs transduced with lentiviral particles expressing either an unrelated RNA (unr), hhRz A7B with the histone stem-loop scaffold (A7B), or the hhRz A7B lacking the scaffold (A7B-w). Cells were selected with puromycin for 72 h and analyzed using anti-CD93 and anti-β-actin antibodies. (G) Quantification of CD93 protein levels from experiments performed as in (F). Values were normalized to β-actin and expressed as percentages relative to control cells (unr). **$p < 0.01$, ****$p < 0.0001$; one-way ANOVA.

hhRzs may be slightly less efficient than RNA interference in silencing target genes,[37] they offer important advantages, including high target specificity and precise cleavage without detectable off-target effects.[21] Notably, hhRz-based gene therapy has already demonstrated safety and biological efficacy, as evidenced by the first hhRz therapeutic agent that advanced to phase II clinical trials for

Table 2. Nucleotide sequences of hhRzs specific for the CD93 mRNA

hhRz	Stem I	Stem II	Stem III	NUH
A4B	4915-GACCTATTTG	HR2	ACAACAAATC-4895	GUC
A6B	4455-CAGGAGAGG	HR2	acCCAGCCT-4436	guc
A7B	3151-GCTTTAAAAT	HR2	ACAACTTTCA-3131	GUC
A9	2747-AAGGCTATTA	HR2	AAATGTAAGA-2727	UUC
A10	3827-AAAACAATCT	HR2	AACCATAGCA-3807	UUC
A11	3947-GTTCCAACT	HR2	*AAA*ACTGGAG-3928	*UUC*
A12	5281-GGCCACC	HR2	aaGGCAGAG-5265	uuc

Stem I and stem III contain annealing sequences designed to target CD93 mRNA, each located adjacent to a NUH triplet and displayed according to an accessibility score color code: underlines indicate high accessibility, italics indicates medium accessibility, and lowercase indicates low accessibility. The last base of the NUH triplet (C) is not shown, as it does not anneal to the hhRz. The sequences are presented in the reverse orientation relative to CD93 mRNA to allow proper base pairing with the target region. Upstream of stem I, the sequence 5′-GGCTCTTCTCAGAGCCAAAAA-3′ was inserted, while downstream of stem III, the sequence 5′-AAAAAGGCTCTTCTCAGAGCCTTTTT-3′ was added and where the stretch of five thymidines serves as a termination signal for RNA polymerase III. Stem II contains the same core sequence as that reported for HR2.

HIV-1 infection.[38] Finally, the success of gene therapy for AMD depends critically on the choice of an appropriate molecular target and its expression profile. CD93 represents an excellent candidate, given its essential role in choroidal neovascularization, its differential expression in healthy and pathological vessels, and the evidence that inhibition of CD93 signaling effectively blocks AMD-associated angiogenesis.[9]

In living cells, however, exogenous RNAs are rapidly degraded by host RNases, and intracellular proteins and compartments can interfere with the diffusion, folding, and target binding of *trans*-cleaving hhRzs.[22] To ensure efficient delivery and activity of *trans*-acting hhRzs in the cellular environment, these molecules must be integrated into RNA scaffolds. Although different scaffolds have been employed to improve structural stability and preserve hhRz activity in living systems,[22] we designed a novel scaffold based on the 3′ stem-loop sequence of canonical histone mRNAs, a structural element essential for histone mRNA stability and regulation.[23] This sequence was incorporated at both the 5′ and 3′ flanking regions of the CD93-specific ribozyme. Unlike other scaffolds,[25] this short histone-derived sequence can be easily inserted into ribozymes without altering their conformation or catalytic activity. Consistent with this design, removal of the stem-loop elements from the best-performing construct markedly reduced ribozyme-mediated silencing, indicating that this scaffold enhances the intracellular performance of the hhRz, most likely by improving its stability in the cellular environment. As a result, the engineered RNA chimera effectively down-regulated CD93 expression and reduced its pro-migratory activity under physiological conditions. These findings open new avenues for the development of ribozyme-based therapeutic tools aimed at controlling pathological angiogenesis. Importantly, the aim of this study was to establish a strategy for the selection of functionally active hhRzs in cells. Further structural optimization of selected ribozymes, including parameters such as antisense flank length that may influence catalytic turnover,[39,40] will be addressed in future studies.

MATERIALS AND METHODS

Computational methods of RNA secondary structure prediction

The secondary structure of the full-length human CD93 mRNA (GenBank accession number NM_012072.4; 6,681 nucleotides in length) was predicted using the RNAfold server from the Vienna

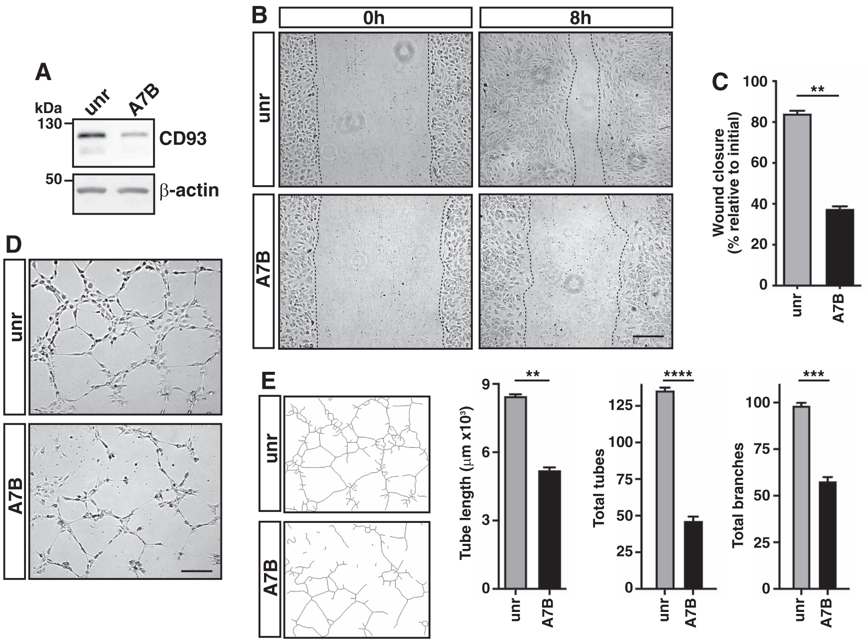

Figure 4. Expression of A7B in ECs impairs cell migration and tubulogenesis

HUVECs were transduced with lentiviral particles expressing either an unrelated (unr) or the A7B hhRbz and selected in puromycin for 24 h. (A) Cell lysates from transduced ECs were analyzed by western blotting using anti-CD93 and anti-β-actin antibodies, the latter serving as a loading control. (B) Representative images of wound healing assays performed with transduced HUVECs. Cells were photographed immediately after scratching (0 h) and after 8 h of incubation. Scale bars, 100 μm. (C) Quantification of wound closure, expressed as the percentage of the initial scratch area covered after 8 h. Data are derived from three independent experiments (n = 3 images per condition per experiment). **$p < 0.01$; paired t test. (D) Representative images of Matrigel tube formation assays performed with transduced HUVECs. Images were taken after 8 h of incubation in complete growth medium. Scale bars, 100 μm. (E) Quantification of total tube length, number of tubes, and number of branching points, as shown in (D). Representative images generated using the Angiogenesis Analyzer plug-in for ImageJ are displayed. **$p < 0.01$, ***$p < 0.001$, and ****$p < 0.0001$; paired t test.

RNA Websuite (http://rna.tbi.univie.ac.at/cgi-bin/RNAWebSuite/RNAfold.cgi). The MFE structure generated by RNAfold was visualized as a two-dimensional representation using the *forna* server (http://rna.tbi.univie.ac.at/forna/).

Vectors and cloning

The full-length human *CD93* cDNA (nucleotides 127–6,231) was cloned into the NheI/XbaI restriction sites of the pcDNA3.1 expression vector and purchased from GenScript (Piscataway, NJ, USA). Two *trans*-acting hhRzs, designated HR1 and HR2, were designed to target the coding region of human CD93 mRNA (Table 1). HR1, HR2, and the catalytically inactive HR2-NC were synthesized by Eurofins Genomics (Ebersberg, Germany). To generate chimeric molecules in which hhRz sequences were flanked by histone stem-loop elements, oligonucleotides corresponding to the sequences listed in Tables 1 and 2 along with their complementary strands were synthesized by Eurofins Genomics. A complete list of all oligonucleotides is provided in Table S1. To generate compatible sticky ends for cloning, forward primers were designed with a 5′ CCGGT overhang and a 3′ G overhang, whereas the reverse primers were designed with a 5′ AATTC overhang and a 3′ A overhang (Table S1). Following phosphorylation and annealing, the double-stranded oligonucleotides were cloned into the AgeI/EcoRI restriction sites of the pLKO.1 lentiviral vector, downstream of the U6 promoter. Sequence accuracy of all constructs was verified by sequencing.

In vitro transcription

Templates for the synthesis of CD93 RNA targets were generated by PCR amplification of a plasmid containing the full-length CD93 sequence using the following primers: A400, 5′-TAATACGACTCAC TATAGGGCTTCAGCTGGGTGGG-3′ (containing the T7 RNA polymerase promoter) and A401, 5′-CTTGCACACGAAGCCCTC-3′, or A400 and A441, 5′-GGTCACCTGACCTGGGC-3′, to generate amplicons corresponding to nucleotides 468–675 and 468–732 of the human CD93 sequence, respectively. The template for ch-HR2 synthesis was obtained by PCR amplification of the lentiviral vector containing the ch-HR2 insert using the following primers: A434, 5′-TAATACGACTCACTATAGGGCTCTTTTCAGAGCCAAAA AG-3′ (containing the T7 RNA polymerase promoter) and A435, 5′-GGCTCTGAAAAGAGCCTTTTTAG-3′. PCR amplicons were purified and used as templates for *in vitro* transcription with the HiScribe T7 High Yield RNA Synthesis Kit (New England Biolabs, Ipswich, MA, USA), according to the manufacturer's instructions. To remove template DNA, the synthesized RNA was treated with DNase I for 15 min at 37°C and subsequently purified using the Monarch RNA Cleanup Kit (New England Biolabs).

In vitro ribozyme cleavage reaction

Cleavage reactions between the hhRzs and the synthetic RNA substrates (208-mer and 265-mer, both containing the cleavage nucleotide C at position 93) were carried out using 100 pmol of ribozyme and 10 pmol of substrate in a total volume of 10 μL. The ribozyme and substrate were first incubated together at 95°C for 1 min in a buffer containing 50 mM Tris-HCl (pH 7.5), 10 mM $MgCl_2$, and 20 mM NaCl, then allowed to cool to room temperature for 10 min and further incubated for 2 h at 37°C. Reactions were stopped by adding an equal volume of RNA Loading Dye (New England Biolabs), followed by heating at 70°C for 5 min and immediate chilling on ice. Reaction products were analyzed by electrophoresis on denaturing 6% TBE-urea gels (Thermo Fisher Scientific, Waltham, MA, USA). Electrophoretic bands were visualized by staining with SYBR Gold (Thermo Fisher Scientific).

Cell cultures and transfection

Primary HUVECs from pooled donors were purchased from Lonza (Walkersville, MD, USA) and cultured on gelatin-coated plates in Endothelial Cell Basal Medium-2 supplemented with growth factors (Lonza), as previously described.[41] Knockdown experiments were performed using pLKO.1 lentiviral vectors expressing either the chimeric ribozymes or an unrelated RNA. Recombinant lentiviruses were produced and used for infection experiments as previously described.[42] After infection, cells were maintained in complete medium containing 4 μg/mL puromycin (Merck, Darmstadt, Germany) to select for stably transduced populations. (HEK293 cells were maintained in DMEM supplemented with 10% fetal bovine serum (FBS) and transfected in culture medium containing 2% FBS and 1 mM sodium butyrate (Merck). Transient transfections of HEK293 cells were carried out using the Transporter 5 Transfection Reagent (Polysciences, Warrington, PA, USA), according to the manufacturer's instructions. For cotransfection experiments, designed to ensure efficient delivery of hhRzs into cells expressing full-length CD93, the hhRz-expressing plasmids and the CD93 expression construct were cotransfected at a 10:1 molar ratio, respectively.

DMS probing of HUVECs and RNA extraction

5×10^6 HUVECs suspended in 500 μL of complete medium were probed with DMS (Merck) at a final concentration of 100 mM. DMS was pre-diluted 1:6 in ethanol prior to use. Cells were incubated with DMS for 2 min at 37°C. The reaction was quenched by adding an equal volume of ice-cold 1 M DTT. Cells were briefly pelleted by centrifugation, the supernatant was discarded, and the pellet was lysed in 1 mL of ice-cold TRIzol Reagent (Thermo Fisher Scientific). RNA samples in TRIzol were mixed with 0.2 volumes of chloroform and vigorously vortexed for 15 s. Samples were incubated at room temperature for 2 min and centrifuged at 12,500 × g for 15 min at 4°C. After phase separation, the upper aqueous phase was transferred to a clean tube, mixed with 2 mL of ethanol, and loaded on Monarch Spin RNA Cleanup columns (New England Biolabs).

Targeted MaP of CD93 and library preparation

For targeted MaPseq of CD93 mRNA, 16 tiling primer pairs were designed using Primer-BLAST,[43] generating amplicons of approximately 450–550 bp. Primer pairs were divided into two non-overlapping sets (odd and even) (Table 3). All reverse primers within each set were pooled equimolarly, and 1 μL of the 20 μM pool was used for reverse transcription of 5 μg of DMS-probed total RNA. Reactions contained 2 μL dNTPs (10 mM each), 4 μL 5× RT Buffer

Table 3. Primer sequences used for targeted DMS-MaPseq analysis

Oligo	Sequence
For-1	AGCTGGGAGCCGAGATAGAA
For-2	CAAGTGCCTGGACCCTAGTC
For-3	TTCCTGTGCAAGGAGAAGGC
For-4	CGAATGCTGGGTTGGCTATG
For-5	CAAGGCTACACCCACCACAA
For-6	ATGCGGCAGACAGTTACTCC
For-7	CCAATTCCGGATCAGGGTGT
For-8	GAGGCAGGAAGTGCCTCTTT
For-9	AACGGGAGATGATGCACTGT
For-10	GAGGCCTCATGTCTCCTGTG
For-11	AAAGGCTGTGTCCATTTGGC
For-12	GGTCATGGAACCCCTCTGTG
For-13	CAGTGGGGAATCCAAGGGTC
For-14	CCAGGGCAGGACAGTTATCG
For-15	TCCCTGCGATCAGGCAAAAA
For-16	GCTTGGGAATCTGAGATATGATCC
Rev-1	GGAGCTCCTTGTGCCAGTTA
Rev-2	CCATACTTGGGGCTGACACA
Rev-3	CGACCCAGAGCACACTCATC
Rev-4	TGAGTGGGGCAGATGTGATG
Rev-5	GTCTCTAGGGCCACCTCACT
Rev-6	TCAGCATGAGCAACTCACCT
Rev-7	CAAATCCCTGGCCTCTCTCG
Rev-8	TGCTGCATCAACTGTGGACT
Rev-9	TGTTCTCCTGCACAGTGGTG
Rev-10	CTCTCCACACCACAGACTGC
Rev-11	GCCAGGGAAGGTTTTGAGGA
Rev-12	GTCACCTGGGCAGAAACACT
Rev-13	GTGGTTGGTCTTGCCTGGAA
Rev-14	AGGGCTTGTGATGAGGATGG
Rev-15	AAACCATGGGTTTTGCAGCC
Rev-16	CTCTGAGCCTTCCCTCCTCA

(250 mM Tris-HCl, pH 8.3; 375 mM KCl; 15 mM $MgCl_2$), 1 μL 0.1 M DTT, 1 μL Induro Reverse Transcriptase (New England Biolabs), and 0.5 μL SUPERase·In RNase Inhibitor (Thermo Fisher Scientific), in a final volume of 20 μL. Following reverse transcription, the RTase-RNA-cDNA complex was disrupted by adding 1 μL of 10 M NaOH and incubating the reaction at 95°C for 3 min. Reactions were purified using Monarch Spin RNA Cleanup columns (New England Biolabs). cDNA was eluted in 20 μL, and 2 μL were used as template for PCR with each primer pair, employing Phusion High-Fidelity DNA Polymerase (New England Biolabs). PCR products from all primer pairs within a set were pooled, resolved on a 2% agarose gel, and a gel slice corresponding to the 450–550 bp region was excised. DNA was purified using the NucleoSpin Gel & PCR Clean-up Mini Kit (Macherey-Nagel, Duren, Germany). Libraries were generated from 10 ng of gel-purified DNA from each set using the NEBNext Ultra II FS DNA Library Prep Kit for Illumina (New England Biolabs), following the manufacturer's instructions.

Analysis of CD93 targeted DMS-MaPseq data

Sequencing reads were aligned to the CD93 mRNA reference sequence using the *rf-map* module of RNA Framework v2.9.3[44] in combination with Bowtie2 v.2.3.5.1,[45] with the following parameters: *-b2 -ctn -cmn 0 -mp "–very-sensitive-local"*. Mutation calling and coverage estimation were performed using the *rf-count* module, providing a mask file containing the primer sequences used to generate each amplicon set. Data from the odd and even primer sets were subsequently merged using the *rf-rctools merge* utility, and normalized DMS reactivities were obtained with the *rf-norm* module. Accessibility scores were computed to identify putative target regions by applying a sliding-window analysis. For each nucleotide, the median DMS reactivity within a 21-nt window centered on that position was calculated, excluding windows with more than 25% missing reactivity values. Accessibility scores were defined as the difference between this local median and the global median reactivity across the entire transcript.

Total RNA extraction and RT-qPCR

Total RNA was extracted from cells using the EuroGold Trifast Reagent (Euroclone, Pero, MI, Italy) according to the manufacturer's instructions. CD93 mRNA expression was evaluated by quantitative reverse transcription PCR using the Luna Universal One-Step RT-PCR Kit (New England Biolabs) and the Rotor-Gene Q thermocycler (Qiagen, Hilden, Germany). The specific primers used for CD93 amplification were: A512, 5′-GTGCATTACAGGTGTTTGTGAAG-3′ and A513, 5′-CCGTAGTCAAGTGTGTGGTAAC-3′.

Immunoblotting

Immunoblotting was performed as previously described.[46] The following primary antibodies were used: mouse monoclonal anti-CD93 (clone 4E1)[3] and mouse monoclonal anti-β-actin (A2228, Merck). Protein band intensities were quantified by densitometric analysis using ImageJ software to compare relative protein expression levels.

Wound healing and Matrigel tube formation assays

The scratch assay was performed as previously described.[12] Briefly, lentivirus-transduced HUVECs were cultured until confluence, and a straight scratch was created in the monolayer using a sterile pipette tip. The cultures were washed and allowed to grow in complete medium. The Matrigel tube formation assay was carried out as reported in a previous study.[5] Exponentially growing lentivirus-transduced HUVECs were detached from culture plates using a non-enzymatic dissociation method[4] and seeded on growth factor-reduced Matrigel (Corning, Corning, NY, USA). Bright-field images of the wound healing and tube formation assays were captured using the Celena S Digital Imaging System (Logos Biosystems, Anyang, South Korea) at multiple positions, and representative fields were

shown. Wound closure (cell migration) and tube formation parameters (total tube length and branching points) were quantified using ImageJ software with the wound healing size tool and the angiogenesis analyzer plug-ins, respectively.

Statistical analysis

Data were analyzed using Prism 9 statistical software (GraphPad, San Diego, CA, USA). Values are expressed as the mean ± SD from at least three independent experiments. Statistical significance between two groups was determined using a two-tailed Student's *t* test, while comparisons among groups were performed using one-way ANOVA followed by Tukey's multiple comparison test. Differences were considered statistically significant at *p* values less or equal to 0.05.

DATA AND CODE AVAILABILITY

The datasets on targeted mutational profiling analysis and sequencing are available from the corresponding author upon reasonable request.[10]

ACKNOWLEDGMENTS

This research was funded by the European Union, Next Generation EU, and MIUR Italia Domani Progetto mRNA Spoke 6 del National Center for Gene Therapy and Drugs based on RNA Technology (CUP B63C22000610006).

AUTHOR CONTRIBUTIONS

Conceptualization, M. Orlandini and D.I.; funding acquisition, M. Olivucci, M. Orlandini, F.G., and G.M.T.; data curation, D.I.; investigation, C.D.P., L.R., S.P., and D.I.; methodology, C.D.P. and D.I.; software and visualization, C.D.P.; writing – original draft, M. Orlandini; writing – review and editing, D.I., F.G., and M. Olivucci.

DECLARATION OF INTERESTS

G.M.T., F.G., and M. Orlandini are founders of UCme Bioscience s.r.l. and members of its scientific advisory board. UCme Bioscience develops new therapeutic solutions for patients affected by neovascular retinopathies.

SUPPLEMENTAL INFORMATION

Supplemental information can be found online at https://doi.org/10.1016/j.omtn.2026.102968.

REFERENCES

1. Carmeliet, P. (2003). Angiogenesis in health and disease. Nat. Med. *9*, 653–660.
2. Cao, Y., Langer, R., and Ferrara, N. (2023). Targeting angiogenesis in oncology, ophthalmology and beyond. Nat. Rev. Drug Discov. *22*, 476–495.
3. Orlandini, M., Galvagni, F., Bardelli, M., Rocchigiani, M., Lentucci, C., Anselmi, F., Zippo, A., Bini, L., and Oliviero, S. (2014). The characterization of a novel monoclonal antibody against CD93 unveils a new antiangiogenic target. Oncotarget *5*, 2750–2760.
4. Barbera, S., Lugano, R., Pedalina, A., Mongiat, M., Santucci, A., Tosi, G.M., Dimberg, A., Galvagni, F., and Orlandini, M. (2021). The C-type lectin CD93 controls endothelial cell migration via activation of the Rho family of small GTPases. Matrix Biol. *99*, 1–17.
5. Galvagni, F., Nardi, F., Maida, M., Bernardini, G., Vannuccini, S., Petraglia, F., Santucci, A., and Orlandini, M. (2016). CD93 and dystroglycan cooperation in human endothelial cell adhesion and migration. Oncotarget *7*, 10090–10103.
6. Lugano, R., Vemuri, K., Barbera, S., Orlandini, M., Dejana, E., Claesson-Welsh, L., and Dimberg, A. (2023). CD93 maintains endothelial barrier function by limiting the phosphorylation and turnover of VE-cadherin. FASEB J. *37*, e22894.
7. Lugano, R., Vemuri, K., Yu, D., Bergqvist, M., Smits, A., Essand, M., Johansson, S., Dejana, E., and Dimberg, A. (2018). CD93 promotes β1 integrin activation and fibronectin fibrillogenesis during tumor angiogenesis. J. Clin. Investig. *128*, 3280–3297.
8. Vemuri, K., de Alves Pereira, B., Fuenzalida, P., Subashi, Y., Barbera, S., van Hooren, L., Hedlund, M., Pontén, F., Lindskog, C., Olsson, A.-K., et al. (2024). CD93 maintains endothelial barrier function and limits metastatic dissemination. JCI Insight *9*, e169830.
9. Tosi, G.M., Neri, G., Barbera, S., Mundo, L., Parolini, B., Lazzi, S., Lugano, R., Poletto, E., Leoncini, L., Pertile, G., et al. (2020). The binding of CD93 to Multimerin-2 promotes choroidal neovascularization. Investig. Ophthalmol. Vis. Sci. *61*, 30.
10. Sun, Y., Chen, W., Torphy, R.J., Yao, S., Zhu, G., Lin, R., Lugano, R., Miller, E.N., Fujiwara, Y., Bian, L., et al. (2021). Blockade of the CD93 pathway normalizes tumor vasculature to facilitate drug delivery and immunotherapy. Sci. Transl. Med. *13*, eabc8922.
11. Khan, K.A., Naylor, A.J., Khan, A., Noy, P.J., Mambretti, M., Lodhia, P., Athwal, J., Korzystka, A., Buckley, C.D., Willcox, B.E., et al. (2017). Multimerin-2 is a ligand for group 14 family C-type lectins CLEC14A, CD93 and CD248 spanning the endothelial pericyte interface. Oncogene *36*, 6097–6108.
12. Galvagni, F., Nardi, F., Spiga, O., Trezza, A., Tarticchio, G., Pellicani, R., Andreuzzi, E., Caldi, E., Toti, P., Tosi, G.M., et al. (2017). Dissecting the CD93-Multimerin 2 interaction involved in cell adhesion and migration of the activated endothelium. Matrix Biol. *64*, 112–127.
13. Orlandini, M. (2025). Cellular role of CD93 and its potential as a future therapeutic target. Expert Opin. Ther. Targets *29*, 179–183.
14. Tosi, G.M., Caldi, E., Parolini, B., Toti, P., Neri, G., Nardi, F., Traversi, C., Cevenini, G., Marigliani, D., Nuti, E., et al. (2017). CD93 as a potential target in neovascular age-related macular degeneration. J. Cell. Physiol. *232*, 1767–1773.
15. Li, Y., Fu, L., Wu, B., Guo, X., Shi, Y., Lv, C., Yu, Y., Zhang, Y., Liang, Z., Zhong, C., et al. (2023). Angiogenesis modulated by CD93 and its natural ligands IGFBP7 and MMRN2: a new target to facilitate solid tumor therapy by vasculature normalization. Cancer Cell Int. *23*, 189.
16. Wang, J.-H., Gessler, D.J., Zhan, W., Gallagher, T.L., and Gao, G. (2024). Adeno-associated virus as a delivery vector for gene therapy of human diseases. Signal Transduct. Targeted Ther. *9*, 78.
17. Amarzguioui, M., and Prydz, H. (1998). Hammerhead ribozyme design and application. Cell. Mol. Life Sci. *54*, 1175–1202.
18. Prody, G.A., Bakos, J.T., Buzayan, J.M., Schneider, I.R., and Bruening, G. (1986). Autolytic processing of dimeric plant virus satellite RNA. Science *231*, 1577–1580.
19. Uhlenbeck, O.C. (1987). A small catalytic oligoribonucleotide. Nature *328*, 596–600.
20. Haseloff, J., and Gerlach, W.L. (1988). Simple RNA enzymes with new and highly specific endoribonuclease activities. Nature *334*, 585–591.
21. Akashi, H., Matsumoto, S., and Taira, K. (2005). Gene discovery by ribozyme and siRNA libraries. Nat. Rev. Mol. Cell Biol. *6*, 413–422.
22. Huang, X., Zhao, Y., Pu, Q., Liu, G., Peng, Y., Wang, F., Chen, G., Sun, M., Du, F., Dong, J., et al. (2019). Intracellular selection of trans-cleaving hammerhead ribozymes. Nucleic Acids Res. *47*, 2514–2522.
23. Marzluff, W.F., Wagner, E.J., and Duronio, R.J. (2008). Metabolism and regulation of canonical histone mRNAs: life without a poly(A) tail. Nat. Rev. Genet. *9*, 843–854.
24. Pley, H.W., Flaherty, K.M., and McKay, D.B. (1994). Three-dimensional structure of a hammerhead ribozyme. Nature *372*, 68–74.
25. Ponchon, L., and Dardel, F. (2007). Recombinant RNA technology: the tRNA scaffold. Nat. Methods *4*, 571–576.
26. Lewin, A.S., and Hauswirth, W.W. (2001). Ribozyme gene therapy: applications for molecular medicine. Trends Mol. Med. *7*, 221–228.
27. Zubradt, M., Gupta, P., Persad, S., Lambowitz, A.M., Weissman, J.S., and Rouskin, S. (2017). DMS-MaPseq for genome-wide or targeted RNA structure probing in vivo. Nat. Methods *14*, 75–82.
28. Yee, E.J., Vigil, I., Sun, Y., Torphy, R.J., Schulick, R.D., and Zhu, Y. (2024). Group XIV C-type lectins: emerging targets in tumor angiogenesis. Angiogenesis *27*, 173–192.
29. Mammadzada, P., Corredoira, P.M., and André, H. (2020). The role of hypoxia-inducible factors in neovascular age-related macular degeneration: a gene therapy perspective. Cell. Mol. Life Sci. *77*, 819–833.

30. Moore, N.A., Bracha, P., Hussain, R.M., Morral, N., and Ciulla, T.A. (2017). Gene therapy for age-related macular degeneration. Expert Opin. Biol. Ther. *17*, 1235–1244.

31. Gragoudas, E.S., Adamis, A.P., Cunningham, E.T., Feinsod, M., and Guyer, D.R.; VEGF Inhibition study in ocular neovascularization clinical trial group (2004). Pegaptanib for neovascular age-related macular degeneration. N. Engl. J. Med. *351*, 2805–2816.

32. Askou, A.L., Pournaras, J.A.C., Pihlmann, M., Svalgaard, J.D., Arsenijevic, Y., Kostic, C., Bek, T., Dagnæs-Hansen, F., Mikkelsen, J.G., Jensen, T.G., and Corydon, T.J. (2012). Reduction of choroidal neovascularization in mice by adeno-associated virus-delivered anti-vascular endothelial growth factor short hairpin RNA. J. Gene Med. *14*, 632–641.

33. Li, C., and Samulski, R.J. (2020). Engineering adeno-associated virus vectors for gene therapy. Nat. Rev. Genet. *21*, 255–272.

34. Ivanova, E., Corona, C., Eleftheriou, C.G., Stout, R.F., Körbelin, J., and Sagdullaev, B.T. (2022). AAV-BR1 targets endothelial cells in the retina to reveal their morphological diversity and to deliver Cx43. J. Comp. Neurol. *530*, 1302–1317.

35. Krolak, T., Chan, K.Y., Kaplan, L., Huang, Q., Wu, J., Zheng, Q., Kozareva, V., Beddow, T., Tobey, I.G., Pacouret, S., et al. (2022). A high-efficiency AAV for endothelial cell transduction throughout the central nervous system. Nat. Cardiovasc. Res. *1*, 389–400.

36. Calton, M.A., Croze, R.H., Burns, C., Beliakoff, G., Vazin, T., Szymanski, P., Schmitt, C., Klein, A., Leong, M., Quezada, M., et al. (2024). Design and characterization of a novel Intravitreal dual-transgene genetic medicine for neovascular retinopathies. Investig. Ophthalmol. Vis. Sci. *65*, 1.

37. Drew, H.R., Lewy, D., Conaty, J., Rand, K.N., Hendry, P., and Lockett, T. (1999). RNA hairpin loops repress protein synthesis more strongly than hammerhead ribozymes. Eur. J. Biochem. *266*, 260–273.

38. Burnett, J.C., and Rossi, J.J. (2009). Stem cells, ribozymes and HIV. Gene Ther. *16*, 1178–1179.

39. Stage-Zimmermann, T.K., and Uhlenbeck, O.C. (1998). Hammerhead ribozyme kinetics. RNA *4*, 875–889.

40. Bertrand, E., Pictet, R., and Grange, T. (1994). Can hammerhead ribozymes be efficient tools to inactivate gene function? Nucleic Acids Res. *22*, 293–300.

41. Orlandini, M., Nucciotti, S., Galvagni, F., Bardelli, M., Rocchigiani, M., Petraglia, F., and Oliviero, S. (2008). Morphogenesis of human endothelial cells is inhibited by DAB2 via Src. FEBS Lett. *582*, 2542–2548.

42. Barbera, S., Nardi, F., Elia, I., Realini, G., Lugano, R., Santucci, A., Tosi, G.M., Dimberg, A., Galvagni, F., and Orlandini, M. (2019). The small GTPase Rab5c is a key regulator of trafficking of the CD93/Multimerin-2/β1 integrin complex in endothelial cell adhesion and migration. Cell Commun. Signal. *17*, 55.

43. Ye, J., Coulouris, G., Zaretskaya, I., Cutcutache, I., Rozen, S., and Madden, T.L. (2012). Primer-BLAST: A tool to design target-specific primers for polymerase chain reaction. BMC Bioinf. *13*, 134.

44. Incarnato, D., Morandi, E., Simon, L.M., and Oliviero, S. (2018). RNA Framework: an all-in-one toolkit for the analysis of RNA structures and post-transcriptional modifications. Nucleic Acids Res. *46*, e97.

45. Langmead, B., and Salzberg, S.L. (2012). Fast gapped-read alignment with Bowtie 2. Nat. Methods *9*, 357–359.

46. Barbera, S., Raucci, L., Lugano, R., Tosi, G.M., Dimberg, A., Santucci, A., Galvagni, F., and Orlandini, M. (2021). CD93 signaling via rho proteins drives cytoskeletal remodeling in spreading endothelial cells. Int. J. Mol. Sci. *22*, 12417.

OMTN, Volume *37*

Supplemental information

Rational design of hammerhead ribozymes as CD93 silencing tools for vascular diseases

Cosimo Damiano Perrone, Luisa Raucci, Sara Papini, Gian Marco Tosi, Federico Galvagni, Massimo Olivucci, Danny Incarnato, and Maurizio Orlandini

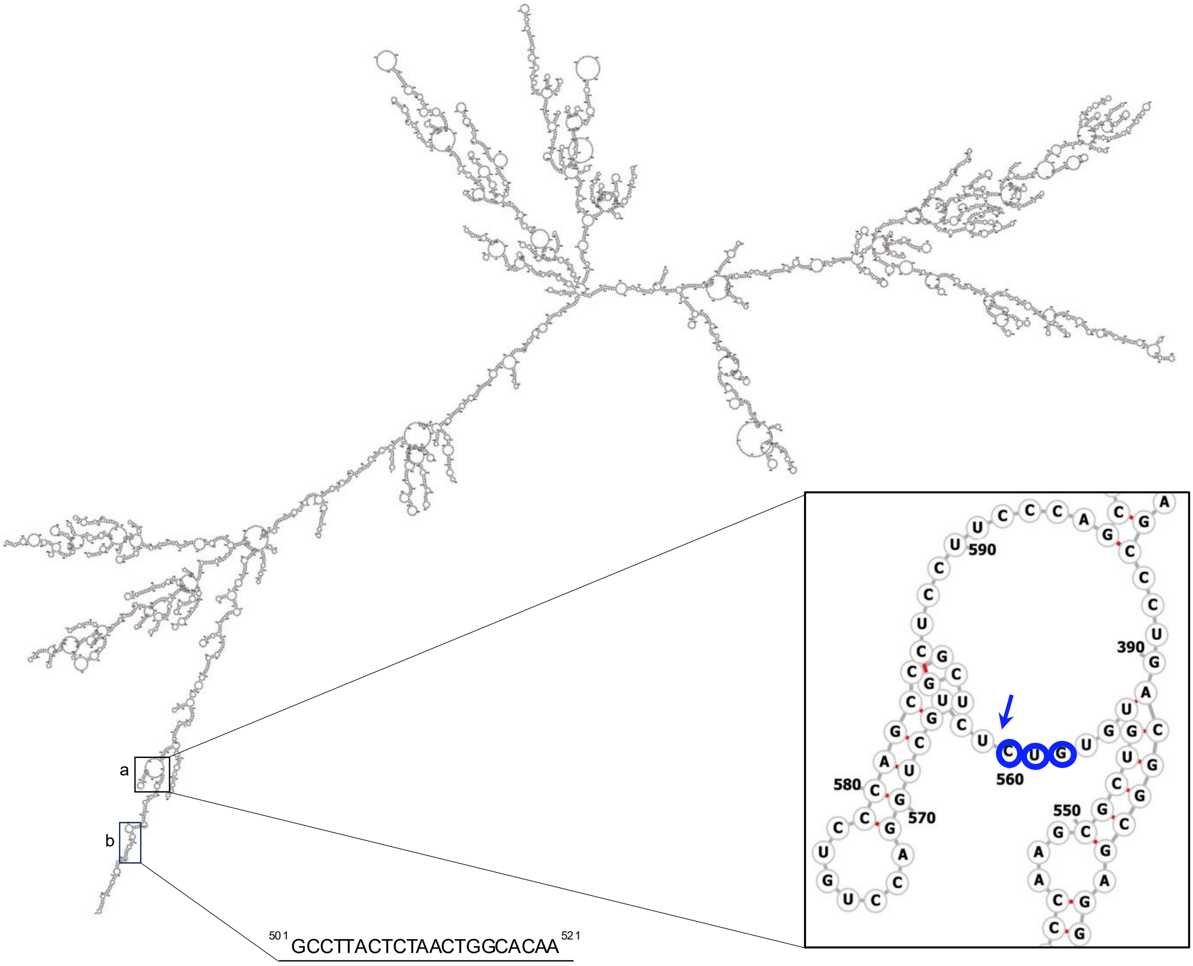

Fig. S1. **Selection of the hhRz target motif within the CD93 mRNA transcript**. The predicted secondary structure (RNAfold) of the full-length CD93 mRNA (6681 nucleotides) is shown. Inset (a) shows the loop containing the selected cleavage site (blue arrow) and the NUH triplet highlighted in blue. Inset (b) displays the CD93 region that pairs with the shRNA (TRCN0000029085) previously used to silence CD93 expression in human ECs.

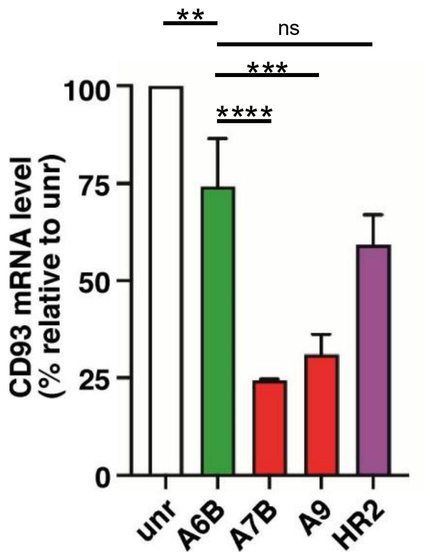

Fig. S2. **RT-qPCR analysis of CD93 mRNA levels following hhRz expression**. Primary HUVECs were transduced with lentiviral particles expressing either an unrelated RNA (unr) or the indicated ch-hhRz construct. Cells were selected with puromycin for 72 h. CD93 mRNA levels were quantified by RT-qPCR, normalized to β-actin, and expressed as percentages relative to control cells (unr). ***P* < 0.01 ****P* < 0.001 *****P* < 0.0001, ns (not significant); one-way ANOVA.

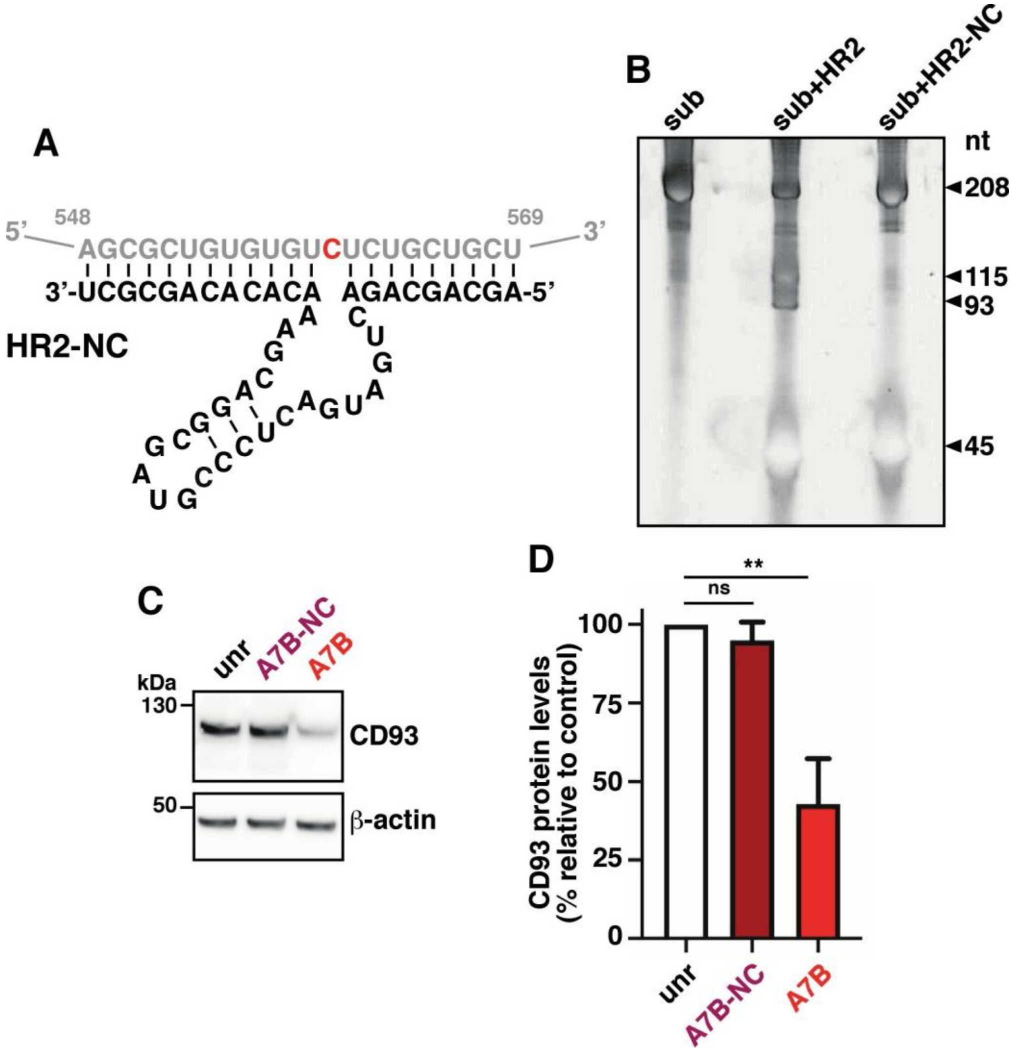

Fig. S3. **Ribozyme-mediated CD93 silencing requires catalytic activity**. A: Secondary structure of the synthetic ribozyme-substrate complex targeting the 558-GUC-560 sequence located within the coding region of CD93 mRNA. HR2-NC carries two mutations in the catalytic core. The cleavage site is indicated in red. Watson-Crick base pairs are shown as dotted lines. B: Denaturing gel electrophoresis showing the cleavage activity of HR2 and HR2-NC on a synthetic CD93 RNA substrate. The substrate (sub) is 208 nucleotides in length and corresponds to nucleotides 468-675 of the full-length CD93 mRNA. Cleavage at the target site (558-GUC-560) generates two fragments of 93 and 115 nucleotides. The control lane shows the untreated RNA substrate (sub). Fragment sizes are indicated in nucleotides (nt). C: HEK 293 cells were co-transfected with a CD93 expression construct and plasmids encoding ch-A7B or ch-A7B-NC, the latter carrying mutations in the catalytic core analogous to HR2-NC. 72 h post-transfection, cell lysates were analyzed by Western blot analysis using anti-CD93 and anti-β-actin antibodies. D: Quantification of CD93 protein levels from experiments performed as in C. Values were normalized to β-actin and expressed as percentages relative to unrelated transfected control cells (unr). $^{**}P < 0.01$, ns (not significant); paired *t*-test.

Table S1. Primer sequences used for hhRz cloning into the pLKO.1 vector.

Oligo	Sequence
A6B fw	ccggtGGCTCTTCTCAGAGCCAAAAACAGGAGAGGCTGATGAGTCCGGTAGCGGACGAAACCCAGCCTAAAAAGGCTCTTCTCAGAGCCTTTTTg
A6B rev	aattcAAAAAGGCTCTGAGAAGAGCCTTTTTAGGCTGGGTTTCGTCCGCTACCGGACTCATCAGCCTCTCCTGTTTTTGGCTCTGAGAAGAGCCa
A4B fw	ccggtGGCTCTTCTCAGAGCCAAAAAGACCTATTTGCTGATGAGTCCGGTAGCGGACGAAACAACAAATCAAAAAGGCTCTTCTCAGAGCCTTTTTg
A4B rev	aattcAAAAAGGCTCTGAGAAGAGCCTTTTTGATTTGTTGTTTCGTCCGCTACCGGACTCATCAGCAAATAGGTCTTTTTGGCTCTGAGAAGAGCCa
A7B fw	ccggtGGCTCTTCTCAGAGCCAAAAAGCTTTAAAATCTGATGAGTCCGGTAGCGGACGAAACAACTTTCAAAAAAGGCTCTTCTCAGAGCCTTTTTg
A7B rev	aattcAAAAAGGCTCTGAGAAGAGCCTTTTTTGAAAGTTGTTTCGTCCGCTACCGGACTCATCAGATTTTAAAGCTTTTTGGCTCTGAGAAGAGCCa
A11 fw	ccggtGGCTCTTCTCAGAGCCAAAAAGTTCCAACTCTGATGAGTCCGGTAGCGGACGAAAAAACTGGAGAAAAAGGCTCTTCTCAGAGCCTTTTTg
A11 rev	aattcAAAAAGGCTCTGAGAAGAGCCTTTTTCTCCAGTTTTTTCGTCCGCTACCGGACTCATCAGAGTTGGAACTTTTTGGCTCTGAGAAGAGCCa
A9 fw	ccggtGGCTCTTCTCAGAGCCAAAAAAAAGGCTATTACTGATGAGTCCGGTAGCGGACGAAAAAATGTAAGAAAAAAGGCTCTTCTCAGAGCCTTTTTg
A9 rev	aattcAAAAAGGCTCTGAGAAGAGCCTTTTTTCTTACATTTTTCGTCCGCTACCGGACTCATCAGTAATAGCCTTTTTTTGGCTCTGAGAAGAGCCa
A10 fw	ccggtGGCTCTTCTCAGAGCCAAAAAAAAACAATCTCTGATGAGTCCGGTAGCGGACGAAAACCATAGCAAAAAAGGCTCTTCTCAGAGCCTTTTTg
A10 rev	aattcAAAAAGGCTCTGAGAAGAGCCTTTTTTGCTATGGTTTTCGTCCGCTACCGGACTCATCAGAGATTGTTTTTTTTTGGCTCTGAGAAGAGCCa
A12 fw	ccggtGGCTCTTCTCAGAGCCAAAAAGGCCACCCTGATGAGTCCGGTAGCGGACGAAAAGGCAGAGAAAAAGGCTCTTCTCAGAGCCTTTTTg
A12 rev	aattcAAAAAGGCTCTGAGAAGAGCCTTTTTCTCTGCCTTTTCGTCCGCTACCGGACTCATCAGGGTGGCCTTTTTGGCTCTGAGAAGAGCCa
HR2 fw	ccggtGGCTCTTCTCAGAGCCAAAAAGCAGCAGACTGATGAGTCCGGTAGCGGACGAAACACACAGCGCTAAAAAGGCTCTTCTCAGAGCCTTTTTg
HR2 rev	aattcAAAAAGGCTCTGAGAAGAGCCTTTTTAGCGCTGTGTGTTTCGTCCGCTACCGGACTCATCAGTCTGCTGCTTTTTGGCTCTGAGAAGAGCCa
A7B-w fw	ccggtGCTTTAAAATCTGATGAGTCCGGTAGCGGACGAAACAACTTTCATTTTTg
A7B-w rev	aattcAAAAATGAAAGTTGTTTCGTCCGCTACCGGACTCATCAGATTTTAAAGCa
A7B-NC fw	ccggtGGCTCTTCTCAGAGCCAAAAAGCTTTAAAATCTGATGACTCCCGTAGCGGACGAAACAACTTTCAAAAAAGGCTCTTCTCAGAGCCTTTTTg
A7B-NC rev	aattcAAAAAGGCTCTGAGAAGAGCCTTTTTTGAAAGTTGTTTCGTCCGCTACGGGAGTCATCAGATTTTAAAGCTTTTTGGCTCTGAGAAGAGCCa
